# Isolation of *Onchocerca lupi* in Dogs and Black Flies, California, USA

**DOI:** 10.3201/eid2105.142011

**Published:** 2015-05

**Authors:** Hassan K. Hassan, Shanna Bolcen, Joseph Kubofcik, Thomas B. Nutman, Mark L. Eberhard, Kelly Middleton, Joseph Wakoli Wekesa, Gimena Ruedas, Kimberly J. Nelson, Richard Dubielzig, Melissa De Lombaert, Bruce Silverman, Jamie J. Schorling, Peter H. Adler, Thomas R. Unnasch, Emily S. Beeler

**Affiliations:** University of South Florida, Tampa, Florida, USA (H.K. Hassan, S. Bolcen, T.R. Unnasch);; National Institute of Allergy and Infectious Diseases, Bethesda, Maryland, USA (J. Kubofcik, T.B. Nutman);; Centers for Disease Control and Prevention, Atlanta, Georgia, USA (M.L. Eberhard);; San Gabriel Valley Mosquito and Vector Control District, West Covina, California, USA (K. Middleton, J.W. Wekesa, G. Ruedas, K.J. Nelson);; University of Wisconsin, Madison, Wisconsin, USA (R. Dubielzig, M. De Lombaert);; Complete Animal Eye Care, Sherman Oaks, California, USA (B. Silverman);; Eye Clinic for Animals, San Diego, California, USA (J.J. Schorling);; Clemson University, Clemson, South Carolina, USA (P.H. Adler);; Los Angeles County Department of Public Health, Los Angeles, California, USA (E.S. Beeler)

**Keywords:** parasites, California, *Onchocerca lupi*, dogs, black flies, zoonoses, filariasis, potcast, United States

## Abstract

In southern California, ocular infections caused by *Onchocerca lupi* were diagnosed in 3 dogs (1 in 2006, 2 in 2012). The infectious agent was confirmed through morphologic analysis of fixed parasites in tissues and by PCR and sequencing of amplicons derived from 2 mitochondrially encoded genes and 1 nuclear-encoded gene. A nested PCR based on the sequence of the cytochrome oxidase subunit 1 gene of the parasite was developed and used to screen *Simulium* black flies collected from southern California for *O. lupi* DNA. Six (2.8%; 95% CI 0.6%–5.0%) of 213 black flies contained *O. lupi* DNA. Partial mitochondrial16S rRNA gene sequences from the infected flies matched sequences derived from black fly larvae cytotaxonomically identified as *Simulium tribulatum*. These data implicate *S. tribulatum* flies as a putative vector for *O. lupi* in southern California.

## Abstract 2

We implicated the black fly as a vector for this filarial zoonotic parasitic infection.

*Onchocerca lupi* is a zoonotic parasite capable of infecting dogs, cats, and humans. Human infection was first suspected in 2002, when a case of human subconjunctival filariasis was found to have a worm with morphology similar to that of *O. lupi* (*1*). Human infection was confirmed in 2011, when a subconjunctival nematode in the eye of a young woman in Turkey was identified by molecular methods as *O. lupi* (*2*). Overall, ≈10 confirmed or suspected human cases have been reported in Turkey (*3*,*4*), Tunisia (*4*), Iran (*5*), the southwestern United States (*6*), Crimea (*1*), and Albania (*1*). In most cases, clinical findings were similar, with a single immature worm found within a periocular mass. In the US case, a mature, gravid female worm was found within a mass in the cervical spinal canal of a young child in Arizona (*6*). The Centers for Disease Control and Prevention recently confirmed 5 additional cases in humans in the southwestern United States (M.L. Eberhard, unpub. data).

Several parasites of the genus *Onchocerca* are known to occur in North America, including 2 in cattle (*O. gutturosa* and *O. lienalis*) (*7*) and 1 in horses (*O. cervicalis*) (*8*). In addition, at least 2 parasites of the native cervid species (*9*) are known to be endemic to North America; at least 1 of these (*O. cervipedis*) has been identified in deer in California (*10*). Although most *Onchocerca* species are associated with ungulates, *O. lupi* is unique in that it is primarily associated with canids. The first report of *O. lupi* infection was in a wolf in Russia (*11*). In the past 20 years, ≈70 cases of *O. lupi* infection have been reported in domestic dogs in the United States, Greece, and Portugal (*12*–*17*). Probable cases also have been reported in Germany, Hungary, Switzerland, and Canada (*16*,*18*,*19*). Many affected dogs contained gravid female worms, presenting the possibility that canids may be a reservoir host for the parasite. The only additional species reported to have been infected were cats: 2 cases were documented in Utah, USA (*20*). Both cats were infected with gravid female worms, suggesting that cats also might be reservoir hosts. However, both cats also were infected with feline leukemia virus and probably were immunosuppressed and therefore not representative of most cats.

In the United States, confirmed and probable *O. lupi* infection has been documented in at least 12 dogs (*17*) and 2 cats (*20*) since 1991. That 6 of the 12 cases in dogs were in southern California (*17*,*21*) highlights this area as a focus of infection. Clinical signs in dogs typically involve 0.3–0.7-cm periocular masses that contain adult worms. Infections may be associated with additional ocular pathology (Figure 1). The masses are typically subconjunctival or episcleral but can be found anywhere in the orbit (*22*).

**Figure 1:**
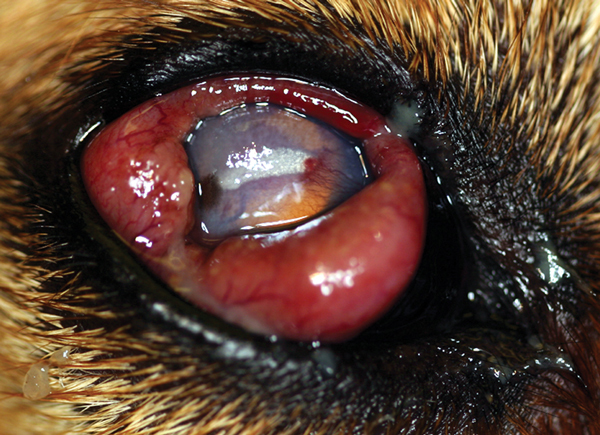
Right eye of a dog with *Onchocerca lupi* infection, southern California, USA, 2004. The dog had severe conjunctival inflammation, corneal degeneration, and an elevated intraocular pressure of 31 mm Hg. Ultimately, enucleation was performed, and histology revealed *Onchocerca* adult worms.

The life cycle of *O. lupi*, including the vector and its primary reservoir host, remains unknown. Determining the vector is the critical step in preventing exposure. Black flies (*Simulium* spp.) and biting midges (*Culicoides* spp.) are vectors for other species of *Onchocerca* (*23*) and might be vectors for *O. lupi*. Black flies are routinely detected in certain areas of Los Angeles County, including a 29-km stretch of the Los Angeles River (http://www.glacvcd.org/), in the San Gabriel Valley area (http://sgvmosquito.org/), and in western areas of the county (http://www.lawestvector.org/). We report 3 additional *O. lupi* infections in dogs in southern California and present molecular evidence implicating the black fly species *S. tribulatum* as the possible vector for this parasite.

## Materials and Methods

### Identification of Cases and Parasites

In Los Angeles County, the Los Angeles County Department of Public Health conducts animal disease surveillance. Private practice veterinarians report diseases in all species, including companion animals. Veterinarians are required to report infectious diseases, particularly those listed as being of priority (http://www.publichealth.lacounty.gov/vet/docs/AnimalReportList2013.pdf), as well as any unusual diseases. 

In March 2012, a local veterinarian reported a case of onchocerciasis in a local dog (dog B). Discussions with the veterinary ophthalmologist and the laboratory examining ocular tissue from the dog revealed an earlier case (dog A) and a concurrent case (dog C).

In May 2006, a 10-year-old, spayed female Labrador Retriever mix (dog A) was examined by a veterinary ophthalmologist in Los Angeles, California. The dog had a brown, lobulated 16-mm episcleral mass in the lateral temporal area of the left eye. One week of topical ophthalmologic antimicrobial and corticosteroid therapy failed to shrink the mass, and it was surgically removed. No other abnormalities were found. The mass contained mixed inflammatory cells surrounding 2 fragments of a cuticle with 2 striae per ridge, characteristic of *O. lupi*. The dog was from the Hollywood Hills area of Los Angeles, ≈3 km south of the 29-km black fly control zone of the Los Angeles River. A travel history was not available.

In February 2012, the same veterinarian examined an 8-year-old female spayed Boxer (dog B). The dog had severe bilateral corneal ulcerations, a 10-mm conjunctival mass in the nasodorsal area of the right eye, and persistent mydriasis in the left eye. No other abnormalities were found. Corneal ulceration is a common disorder in Boxers, but the mass was considered to be unrelated to the ulcers (*24*). The mass was surgically removed and the ulcers treated surgically and medically. The mass contained multifocal granulomatous nodules with central necrosis surrounding sections of partially mineralized nematodes with 2 striae per ridge, characteristic of *O. lupi*. The dog lived in the San Fernando Valley area of Los Angeles, within 5 km of the black fly control zone of the Los Angeles River. It had lived in the Los Angeles area all of its life.

In January 2012, a 4-year-old pit bull mix (dog C) was examined by a veterinary ophthalmologist in San Diego. The dog had 2 episcleral masses (10 mm and 5 mm) at the lateral limbus of the left eye. The masses were immediately adjacent to each other and were associated with the lateral rectus muscle. No other abnormalities were found. The masses were surgically removed, and *O. lupi* was identified morphologically in the tissues. The dog was living at a humane society in San Diego, and further history on the dog was not available.

### Molecular Characterization of Parasite Samples

Unstained, formalin-fixed, paraffin-embedded tissues containing the parasite were obtained from each of the 3 dogs. DNA was extracted by using a kit designed for purification of DNA from formalin-fixed paraffin-embedded tissue (QIAamp DNA FFPE Tissue Kit; QIAGEN, Valencia, CA, USA) according to the manufacturer’s instructions. DNA was then amplified by using each of 3 sets of primers targeting highly conserved nematode or filarial species. This set included primers for the internal transcribed spacer (ITS) in the 5S rRNA gene: (S2: 5′-GTTAAGCAACGTTGGGCCTGG-3′; S16: 5′-TTGACAGATCGGACGAGATG-3′) the 12S small subunit rRNA gene of the mitochondrion (12SF: 5′-GTTCCAGAATAATCGGCTA-3′, 12SR: 5′-ATTGACGGATGRTTTGTACC-3′), and cytochrome oxidase I (*CO*I; COIF: 5′-TGATTGGTGGTTTTGGTAA-3′, COIR: 5′-ATAAGTACGAGTATCAATATC-3′), as described previously (*25*,*26*). PCR products were cloned into the TOPO-TA plasmid vector (Life Technologies, Grand Island, NY, USA) according to the manufacturer's instructions, and plasmids were prepared and sequenced in both directions by using TOPO-specific primers as described previously through a commercial service (Macrogen, Gaithersburg, MD, USA). DNA sequences were initially analyzed using BLAST (http://blast.ncbi.nlm.nih.gov/Blast.cgi). Sequence alignments were performed using ClustalW (*27*), and an unrooted phylogenetic reconstruction was performed using the neighbor-joining method followed by bootstrap analysis using the MegAlign V11 routine of the DNAStar program package (DNAStar, Madison, WI, USA). Sequences were submitted to GenBank with the following accession numbers: KC763786, KC763785, KC763784, KC763783, KC763782, KC763781, KC763780, KC763779, and JX489168.

### Black Fly Collection and Processing

During April–August 2013, we collected 248 black flies from 13 locations in the San Gabriel Valley in Los Angeles County throughout an area ≈380 km^2^. This area is ≈40–50 km east of the veterinary clinic that diagnosed *O. lupi* infection in dogs A and B and ≈180 km north of the clinic that diagnosed it in dog C. The area contains the watershed of the San Gabriel River. The convenience sample of black flies was caught in CO_2_-baited encephalitis virus surveillance traps that had been set for mosquito collection. Each fly was identified as belonging to the *Simulium* genus by standard taxonomic keys and was fixed in 70% ethanol.

We prepared DNA from the individual flies by using the DNeasy Blood and Tissue Kit (QIAGEN) following the manufacturer’s instructions. Flies were prepared in batches of 12 samples; each batch contained 10 individual flies and 2 sham extractions that served as negative controls. A total of 2 μL of the purified genomic product was then used as a template in a nested PCR targeting the *O. lupi* *CO*1 gene. All PCR reactions were conducted in a total volume of 50 μL. The initial amplification reaction was conducted in a solution of 300 mmol/l Tris-HCL (pH 9.0); 75 mmol/l (NH_4_)_2_SO_4_; 10 mmol/l MgCl_2_; 200 μmol/l each of dATP, dCTP, dGTP, and dTTP; 0.5 μM of each primer, and 2.5 U of Taq DNA polymerase (Invitrogen). Primer sequences employed in the initial reaction were 5′-TGTTGCCTTTGATGTTGGGG-3′ and 5′ GGATGACCGAAAAACCAAAACAAG-3′, and amplification conditions were 94°C for 3 min, followed by 35 cycles of 94°C for 45 s, 52°C for 30 s, and 72°C for 90 s, with a final extension of 72°C for 10 min. This reaction produced an amplicon of 475 bp. A total of 1 μL of the product of the first reaction was used as the template in the nested reaction, which used the buffer conditions described above and primers with the sequences 5′-TCAAAATATGCGTTCTACTGCTGTG-3′ and 5′-CAAAGACCCAGCTAAAACAGGAAC-3′. Cycling conditions consisted of 94°C for 4 min, followed by 40 cycles of 94°C for 45 s, 50°C for 45 s, and 72°C for 90 s, and a final extension of 72°C for 10 min. This reaction produced an amplicon of 115 bp. Products from the nested reaction were analyzed by electrophoresis on a 1.5% agarose gel. Samples producing a band of the appropriate size in the initial screens (115 bp) were subjected to a second independent PCR. Samples producing products of the expected size were considered as putative positives. Amplicons of putative positives were subjected to DNA sequence analysis to confirm the identity of the product, using a commercial service (Genewiz, South Plainfield, NJ, USA). We calculated 95% CIs surrounding the estimate of the proportion of infected flies using standard statistical methods (*28*).

### Fly Identification

We amplified a portion of the mitochondrial 16S rRNA gene from the DNA prepared from the infected flies, following previously published protocols (*29*). The primers used in the amplification reaction were 16S F: 5′-CGCCTGTTTATCAAAAACAT-3′ and 16S R: 5′-CTCCGGTTTGAACTCAGATC-3′. The resulting amplicons were subjected to DNA sequence analysis, as described above. The DNA sequences obtained were submitted to the GenBank sequence database under accession numbers KP233211 and KP233212.

Black fly larvae were collected from 4 sites near the locations where the infected flies were trapped. The isolated larvae were cut in half horizontally immediately upon collection. The anterior end of each larva (head) was fixed in 70% isopropanol (rubbing alcohol), and the posterior end (abdomen) was fixed in Carnoy’s solution (3 parts 95% ethanol and 1 part glacial acetic acid by volume).

DNA was prepared from the heads of the fixed larvae and used to amplify the portion of the mitochondrial 16S rRNA gene, as described above. The abdomen of each larva was opened ventrally with fine needles and stained with the Feulgen method (*30*). Salivary glands with stained nuclei and 1 gonad for sex determination were dissected from the abdomen, placed in a drop of 50% acetic acid, flattened under a coverslip, and examined with oil immersion. Identifications were based on diagnostic species-specific rearrangements of the polytene chromosomes (*31*,*32*).

## Results

**Figure 2:**
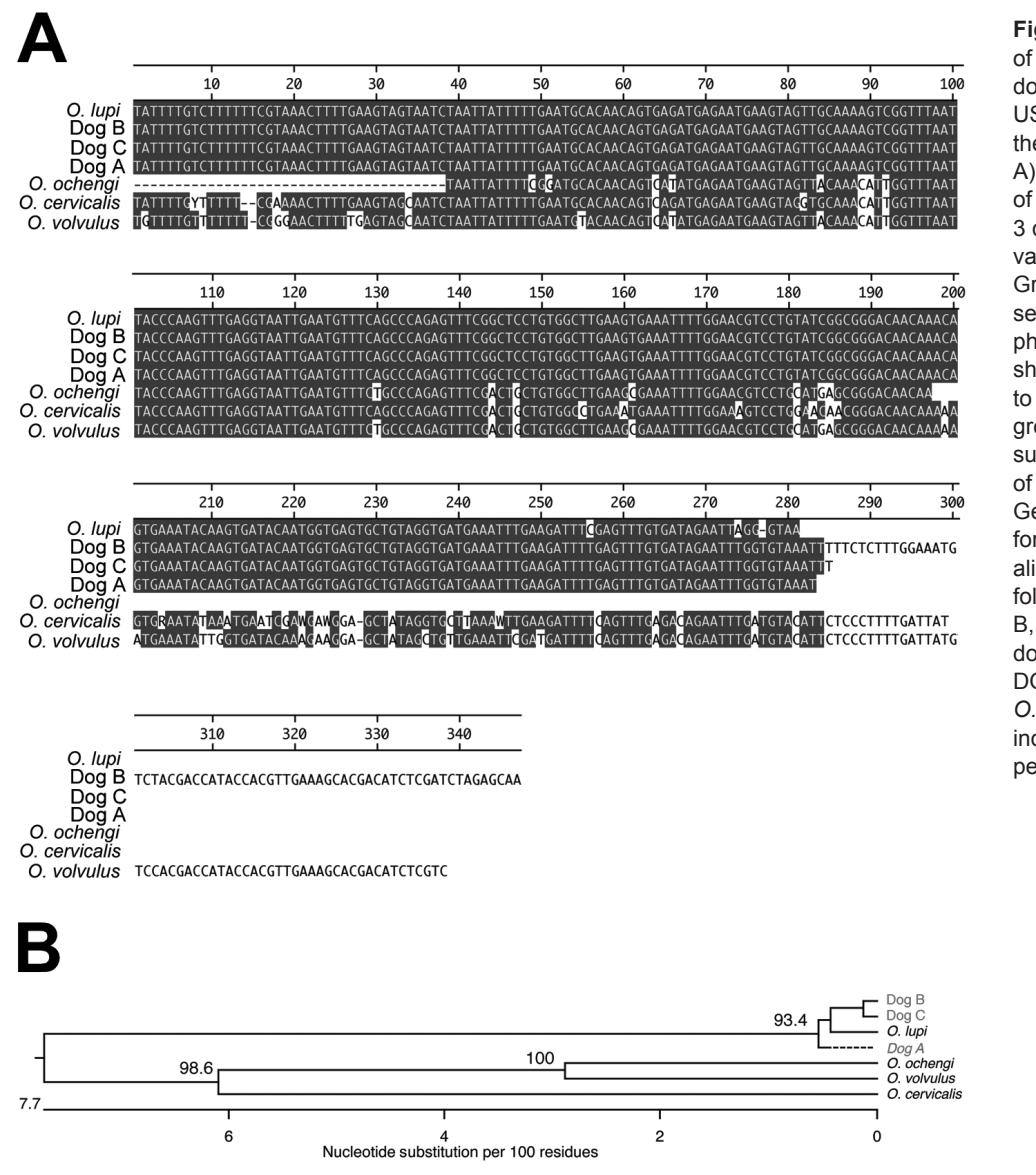
Molecular identification of parasites collected from 3 dogs in southern California, USA, by using sequence from the rRNA internal spacer (ITS). A) Multiple sequence alignment of ITS sequences from the 3 dogs and sequences from various *Onchocerca* parasites. Gray shading indicates areas of sequence identity. B) Unrooted phylogeny of the sequences shown in panel A. Numbers refer to the percentage of times the grouping distal to the number was supported in a bootstrap analysis of 1,000 replicate datasets. GenBank accession numbers for the sequences used in the alignment and phylogeny were as follows: *O. lupi*, JX489168; dog B, KC763782; dog C, KC763781; dog A, KC763779; *O. ochengi*, DQ523781; *O. cervicalis*, U13678; *O. volvulus*, AF325546. Scale bar indicates nucleotide substitutions per 100 residues.

Unstained, formalin-fixed, paraffin-embedded tissue from the 3 dogs was used to amplify parts of 2 mitochondria-encoded (*CO*1 and 12S rRNA) and 1 nuclear-encoded (rRNA ITS1) genes. On the basis of the alignments and phylogenetic analyses, each parasite isolated from the 3 dogs was shown unequivocally to be *O. lupi*. For example, the sequences obtained from the ITS1 amplicon from the parasites from each dog were close to 100% identical to an *O. lupi* isolate from Hungary (Figure 2). Identical relationships were obtained when the 12S rDNA and CO1 PCR amplicons were analyzed (Technical Appendix Figures 1, 2).

**Figure 3:**
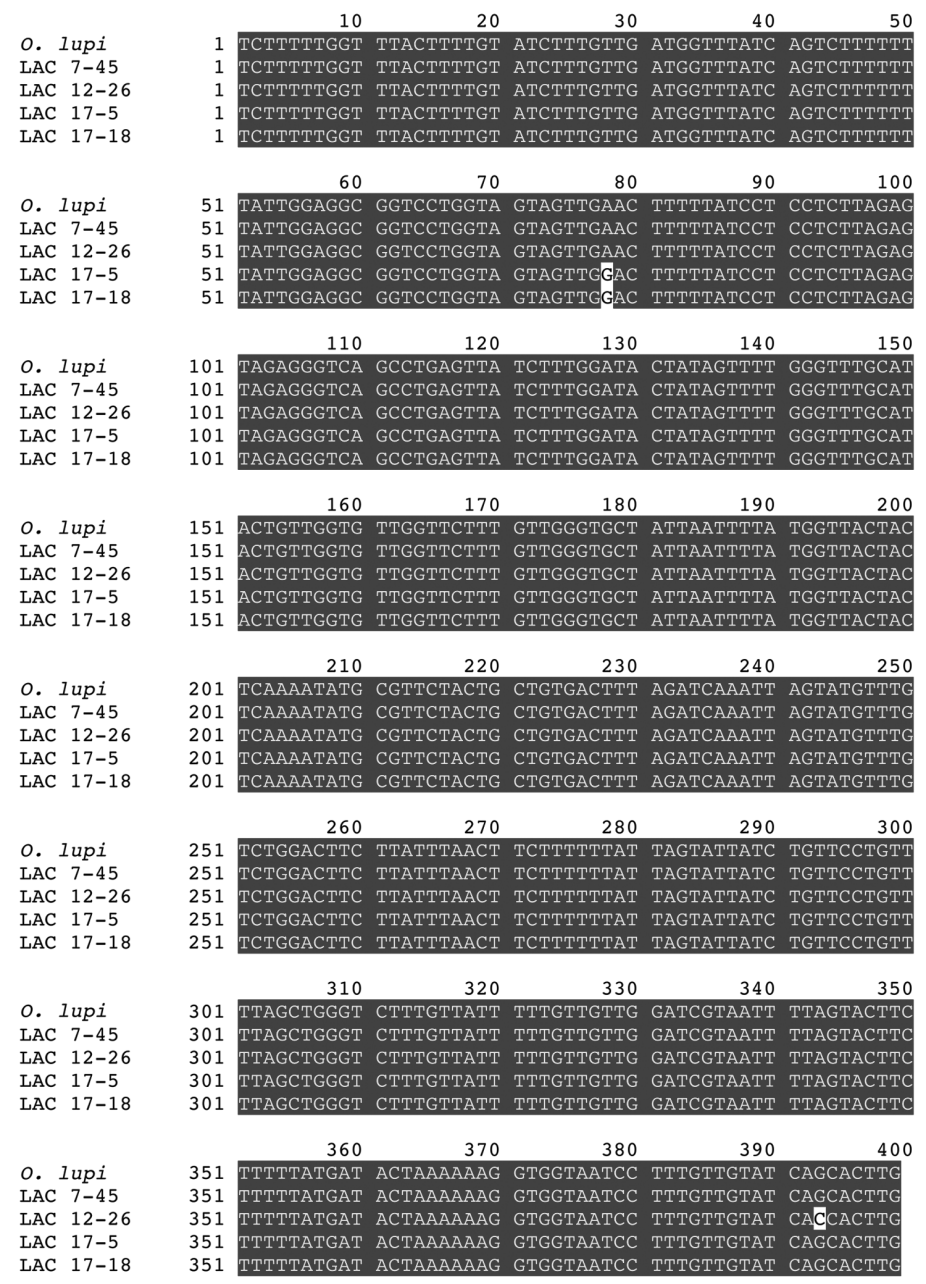
Sequence of the *Onchocerca lupi* cytochrome oxidase subunit 1 gene amplicons recovered from infected flies, southern California, USA, 2012. Gray shading indicates areas of sequence identity.Labels refer to the sample number of the individual infected files from which the sequences were obtained. *O. lupi* GenBank accession no. KC763786. LAC, Los Angeles County.

Of the 248 individual black flies collected, 213 were screened using the nested PCR targeting the *O. lupi* *CO*1 gene. Of these, 6 (2.8%; 95% CI 0.6%–5.0%) produced nested amplicons of the expected size of 115 bp. The sequences of all 6 amplicons exactly matched the published *O. lupi* *CO*1 sequence (data not shown). We then attempted to recover the amplicon from the first reaction and determine the DNA sequence of this larger fragment. This attempt was successful in 4 of the 6 positive flies, resulting in 399 bp of sequence between the primer sites. The sequence of the recovered amplicons matched that of the GenBank reference sequence almost exactly in all 4 samples (Figure 3). We noted single-nucleotide polymorphisms in 3 of the 4 amplicons when we compared them with published sequence; 2 of the isolates shared 1 polymorphism (Figure 3).

Of the 13 locations sampled, 5 contained positive flies (Technical Appendix Figure 3); 1 location had 2 positive flies (Table). These 5 locations spanned ≈270 km^2^, covering most of the sampled area, except its northwest corner. Of the 5 positive sites, 4 were within a circle with a radius of 17.5 km (Technical Appendix Figure 3). Three of the 5 positive collection sites were located along the San Gabriel River (Technical Appendix Figure 3). All positive flies were collected during the spring (April 22–June 4, 2013).

**Table:** Species identification of *Onchocerca lupi* –infected flies, southern California, USA, 2012

Infected fly no.	Collection date	Location	Latitude	Longitude	Species and genotype*
LAC 3-2	2013 Apr 22	Monterey Park City Yard	34.05341	−118.116537	*Simulium tribulatum* A
LAC 5-1	2013 Apr 24	Bernard Biostation	34.116667	−117.7125	*S. tribulatum* A
LAC 7-45	2013 Apr 29	Walnut Coop	34.029101	−117.854616	*S. tribulatum* A
LAC 12-26	2013 May 7	Rainbow Canyon Ranch	34.144731	−117.935686	ND
LAC 17-5	2013 Jun 4	Santa Fe Dam	34.116667	−117.95	*S. tribulatum* B
LAC 17-18	2013 Jun 4	Santa Fe Dam	34.116667	−117.95	*S. tribulatum* B

*LAC, Los Angeles County; ND, definitive identification not successful.

To determine the identity of the infected flies, we amplified a portion of the black fly mitochondrial 16S rRNA gene from the remaining DNA samples. This sequence has previously been shown to be phylogenetically informative in distinguishing several North American black fly species (*29*). A comparison of the sequence data obtained from the amplicons with the GenBank sequence database showed that the sequences were most similar to members of the genus *Simulium*; however, an exact match was not obtained to any of the sequences in GenBank, which precluded identification of the infected flies to the species level (data not shown). To identify the flies to the species level, we collected black fly larvae from sites near the locations from which the infected flies were trapped (Technical Appendix Figure 3). These larvae were bisected and preserved for molecular and cytotaxonomic analyses. The diagnostic portion of the 16S rRNA gene was then amplified from the collected larvae and compared with the sequences obtained from the infected flies. Larvae that had sequences matching those of the infected flies exactly were then identified by cytotaxonomy. Of the 6 infected flies, 5 were identified as *S. tribulatum* using this process (Table). Two 16S mitochondrial alleles were identified in the population of larvae and infected flies identified as *S. tribulatum*. These were designated *S. tribulatum* A and *S. tribulatum* B, which were 97% similar to one another (Technical Appendix Figure 4). *S. tribulatum* B was identified in the infected flies from the Santa Fe Dam, whereas infected flies from the Monterey Park City Yard, Bernard Biostation, and Walnut Coop contained *S. tribulatum* A (Table). The sequence of the infected fly from Rainbow Canyon Ranch matched that of 1 larva in the collection; however, definitive cytotaxonomic identification of this larva was not successful because of poor fixation.

## Discussion

Our data imply that *O. lupi* infection in dogs is ongoing in southern California. The possibility that dogs might be serving as sentinels for this infection suggests that humans and cats in the area also could be at risk for infection.

Several other *Onchocerca* species are endemic to North America. However, except for *O. lupi*, all of these are known to use ungulates as their primary hosts. Thus, isolation of these parasites from dogs, together with the phylogenetic analysis of 3 different gene sequences that all group the isolates with *O. lupi*, strongly support the identification of these parasites as *O. lupi*.

The nested PCRs detected *CO*1-derived amplicons with sequences 99.5%–100% identical to the published *O. lupi* *CO*1 sequence in 6 flies. Some of these sequences could be derived from other *Onchocerca* because *Simulium* spp. flies are known to be vectors for several *Onchocerca* species for which sequence data are not available (*33*,*34*). However, previous studies have suggested that sequence variation in the mitochondrial genome varies from 7% to 15% among *Onchocerca* species (*9*,*35*), and intraspecies variation within the mitochondrial genome is limited in the genus *Onchocerca* (*9*,*35*). Therefore, the sequences detected in the flies are unlikely to have derived from a species other than *O. lupi*.

Our data suggest that the black flies collected frequently fed on a host species that was infected with *O. lupi*, a host that remains unidentified. However, our data implicate *S. tribulatum* flies as the vector for *O. lupi* in southern California. *S. tribulatum*, a member of the *S. vittatum* species complex, is one of the most abundant and widespread species of *Simulium* flies in North America (*36*). *S. tribulatum* flies generally feed on large mammals (e.g., cattle or horses) and rarely bite humans (*36*).

The implication of *S. tribulatum* flies as a possible vector for *O. lupi* also might provide insight about the reasons that *O. lupi* cases have primarily been found in the southwestern United States. Cities and human settlements there typically rely on anthropogenic water sources, such as aquifers, reservoirs, and other water impoundments. The *S. vittatum* complex (of which *S. tribulatum* is a member) includes some of the few black fly species in North America that prosper in these environments (*36*).

The assay we used to detect *O. lupi* in the black flies cannot distinguish between viable and nonviable parasites or immature and infective larvae. Thus, although our data implicate *S. tribulatum* flies as the vector, additional studies are needed to confirm this hypothesis. Laboratory colonies of *S. vittatum*, a sibling species of *S. tribulatum*, could prove useful in confirming that flies of this species complex are actually competent vectors for this parasite (*37*).

The black flies that tested positive for *O. lupi* came from geographic locations adjacent to the San Gabriel River and its watershed. During the past 20 years, southern California has tried to restore natural watershed and wetland habitats, including those in the San Gabriel Valley area (*38*). Black flies rely on rivers and other bodies of water, often with aquatic vegetation for egg laying and larval development, all of which the San Gabriel River and Los Angeles River watersheds provide.

The San Gabriel Mountains are directly upstream of the sites from which we collected the larvae and positive flies. The San Gabriel River, its watershed, and its recreational areas are likely to be providing a wildlife corridor that enables an easily accessible transmission interface. Although most cases in canids have been described in domestic dogs, the relative rarity of infections in domestic animals suggests that the parasite uses a different species as its primary reservoir. The ubiquitous presence of coyotes and other nondomestic canids in the San Gabriel watershed might provide a convenient natural reservoir for the parasite. Additional studies involving sampling of the coyote population in the area, coupled with molecular identification of the blood meals taken by the local black flies (*39*), would be useful in resolving these questions.

Prevention of *O. lupi* infection ultimately might rely on effective *Simulium* control programs, which must address black fly breeding in a variety of settings. The most effective control methods used for the past 20 years in the San Gabriel Valley have been applications of VectoBac 12AS (*Bt*i) (K. Fujioka, San Gabriel Valley Mosquito and Vector Control District, pers. comm.) and occasionally stopping the flow of water for a minimum of 48 hours because the larvae are vulnerable to desiccation (*40*). The role of ivermectin, milbemycin, and other heartworm preventive medications commonly used in dogs and cats is unknown. These medications would probably kill microfilariae, but their efficacy against infective L3 larvae of *O. lupi* is unknown. These medications in pets may play a role in preventing infection or in preventing infected pets from serving as reservoir hosts, reducing transmission of this infection.

Technical Appendix Molecular identification of parasites collected from dogs by using sequences derived from the mitochondrial 12s rRNA gene; molecular identification of parasites collected from dogs by using sequences derived from the cytochrome oxidase subunit 1 gene; map of San Gabriel Valley, Los Angeles County, California, USA, showing locations where adult black flies and black fly larvae were collected during 2012; and sequence of the 2 alleles obtained from amplification of a portion of the mitochondrial 16s rRNA gene from *Simulium tribulatum* infected with *Onchocerca lupi*.
