# Supplementary material for: Isolation of Onchocerca lupi in Dogs and Black Flies, California, USA
Source: Emerg Infect Dis. 2015 May;21(5):789–96. doi: 10.3201/eid2105.142011 (PMC4412245; doi:10.3201/eid2105.142011)
Supplement: Technical Appendix — Molecular identification of parasites collected from dogs by using sequences derived from the mitochondrial 12s rRNA gene; molecular identification of parasites collected from dogs by using sequences derived from the cytochrome oxidase subunit 1 gene; map of San Gabriel Valley, Los Angeles County, California, USA, showing locations where adult black flies and black fly larvae were collected during 2012; and sequence of the 2 alleles obtained from amplification of a portion of the mitochondrial 16s rRNA gene from Simulium tribulatum infected with Onchocerca lupi. [file 14-2011-Techapp-s1.pdf]

Article DOI: http://dx.doi.org/10.3201/eid2105.142011

Isolation of *Onchocerca lupi* in Dogs and Black Flies, California, USA

Technical Appendix

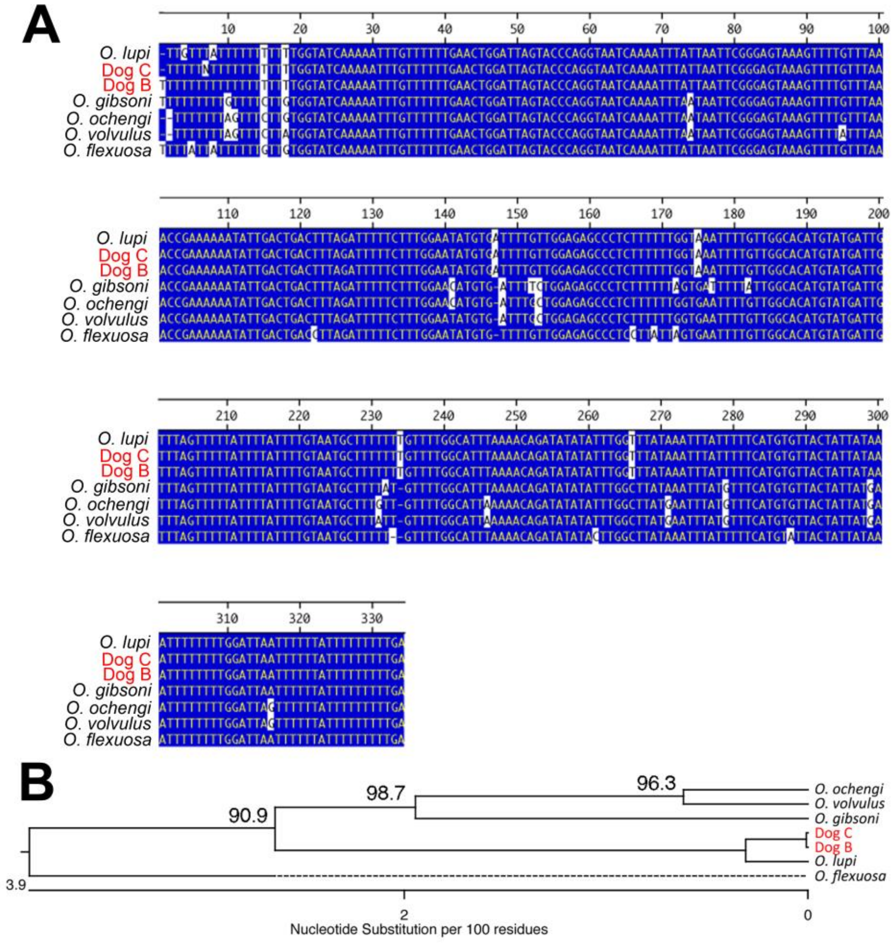

Technical Appendix Figure 1. Molecular identification of parasites collected from dogs by using sequences derived from the mitochondrial 12s rRNA gene. A) Multiple sequence alignment of 12s rRNA sequences from the 3 dogs described in the text and sequences from various *Onchocerca* parasites. B) Unrooted phylogeny of the sequences shown in panel A. Numbers refer to the percentage of times that the grouping distal to the number was supported in a bootstrap analysis of 1,000 replicate datasets. GenBank accession numbers for the sequences used in the alignment and phylogeny were as follows: *O. lupi*, GU365879; dog C, KC763783; dog B, KC763784; *O. gibsoni*, AY462913; *O. ochengi*, DQ523741; *O. volvulus*, DQ523741; *O. flexuosa*, HQ214004.

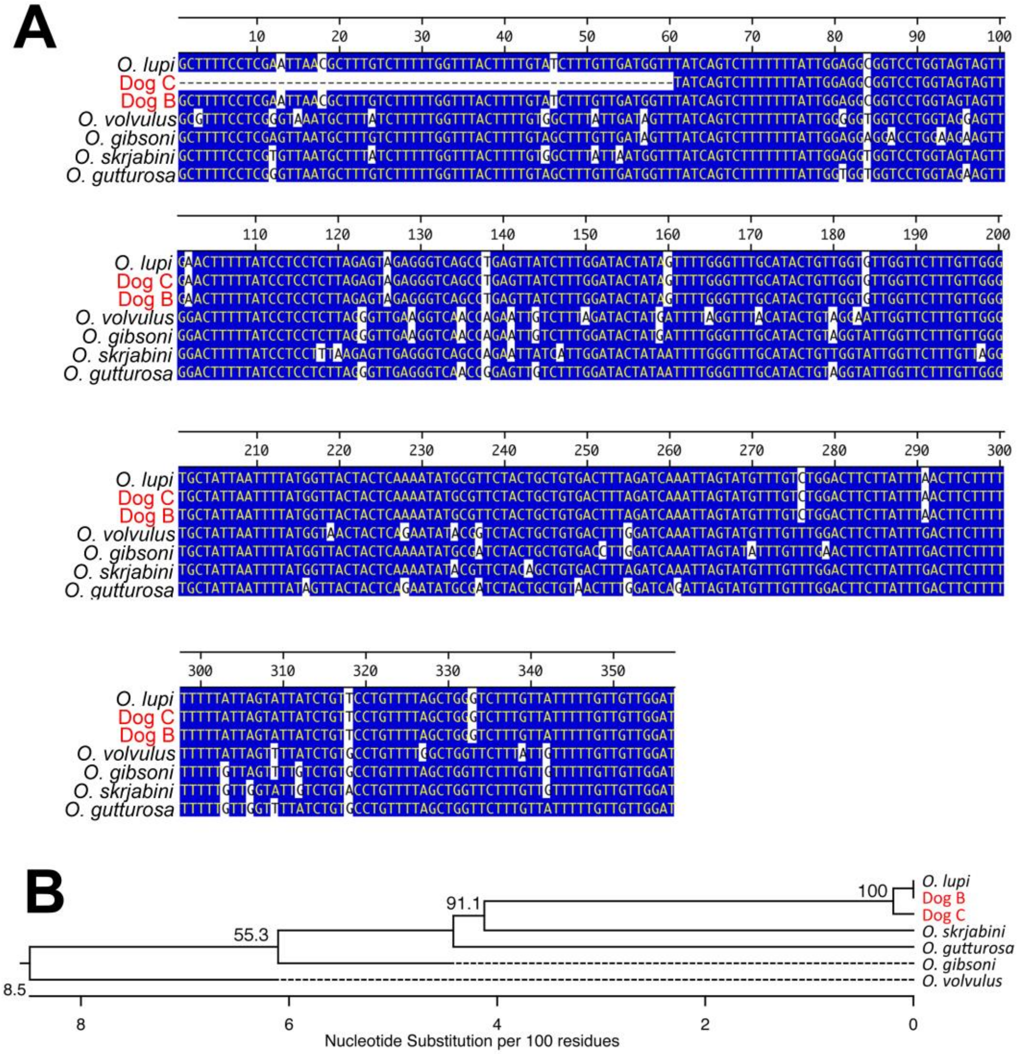

Technical Appendix Figure 2. Molecular identification of parasites collected from dogs by using sequences derived from the cytochrome oxidase subunit 1 (*CO*1) gene. A) Multiple sequence alignment of *CO*1 sequences from the 3 dogs described in the text and sequences from various *Onchocerca* parasites. Blue shading indicates areas of sequence identity. B) Unrooted phylogeny of the sequences shown in panel A. Numbers refer to the percentage of times the grouping distal to the number was supported in a bootstrap analysis of 1,000 replicate datasets. GenBank accession numbers for the sequences used in the alignment and phylogeny were as follows: *O. lupi*, JX080031; dog C, KC763785; dog B, KC763786; *O. volvulus*, AF0151931; *O. skrjabini*, AM749271; *O. gutturosa*, AJ271617.

```
                              10         20         30         40         50
S. tribulatum A    1 CCGCAGTATT TTGACTGTGC AAAGGTAGCA TAATCATTAG TCTTTTAATT    50
S. tribulatum B    1 CCGCAGTATT TTGACTGTGC AAAGGTAGCA TAATCATTAG TCTTTTAATT    50

                              60         70         80         90        100
S. tribulatum A   51 GAAGGCTGGT ATGAATGGTT GGATGAGGTA CAAGCTGTGT CATAAAAATT   100
S. tribulatum B   51 GAAGGCTGGT ATGAATGGTT GGATGAGGTA CAAGCTGTGT CATAAAAATT   100

                             110        120        130        140        150
S. tribulatum A  101 AATATTTGAA TTTAACTTTT TAGTCAAAAG GCTAAAATGT AATTAAAAGA   150
S. tribulatum B  101 AATAATTGAA TTTAACTTTT TAGTCAAAAG GCTAAAATTT AATTAAAAGA   150

                             160        170        180        190        200
S. tribulatum A  151 CGAGAAGACC CTATAGAGCT TTATATAGTT GATATTTAAT TTATTAAGAT   200
S. tribulatum B  151 CGAGAAGACC CTATAGAGCT TTATATTACA AATATTTAAT TTATTAAGAT   200

                             210        220        230        240        250
S. tribulatum A  201 TTATTAAATT TAATTATTTT ACTGTATTTT GTTGGGGTGA CAATAAAATT   250
S. tribulatum B  201 TTATTAAAAT TAATTATTTT GTTTTATTTT GTTGGGGTGA CAATAAAATT   250

                             260        270        280        290        300
S. tribulatum A  251 TATAAAACTT TTATTATTAT TTAACATTTA TTTATGGTTA TATGATCCAG   300
S. tribulatum B  251 TATAAAACTT TTATTAATAT TTAACATTTA TTTATGATTA TATGATCCAG   300

                             310        320        330        340        350
S. tribulatum A  301 TTTTATTGAT TATAAATTTA AGTTACCTTA GGGATAACAG CGTAATTTTT   350
S. tribulatum B  301 TTTTATTGAT TATAAATTTA AGTTACCTTA GGGATAACAG CGTAATTTTT   350

                             360        370        380        390        400
S. tribulatum A  351 TTTGAGAGTT CATATCGACA AAAAAGATTG CGACCTCGAT GTTGGATTAA   400
S. tribulatum B  351 TTTGAGAGTT CATATCGACA AAAAAGATTG CGACCTCGAT GTTGGATTAA   400

                             410        420        430        440        450
S. tribulatum A  401 GAGTAATTTT GGGTGTAGAA GTTCAAAGTT TAAGTCTGTT CGACTTTTAA   450
S. tribulatum B  401 GAGTAATTTT GGGTGTAGAA GTTCAAAGTT TAAGTCTGTT CGACTTTTAA   450

                             460        470        480        490        500
S. tribulatum A  451 ATTCTTACAT .......... .......... .......... ..........   500
S. tribulatum B  451 ATTCTTACAT .......... .......... .......... ..........   500
```

Technical Appendix Figure 3. Map of San Gabriel Valley in Los Angeles County, California, showing locations where adult black flies (black circles) and black fly larvae (black diamonds) were collected during 2012. Boxes indicate sites where flies were positive for *Onchocerca lupi*.

```
                                 10         20         30         40         50
S. tribulatum A     1 CCGCAGTATT TTGACTGTGC AAAGGTAGCA TAATCATTAG TCTTTTAATT      50
S. tribulatum B     1 CCGCAGTATT TTGACTGTGC AAAGGTAGCA TAATCATTAG TCTTTTAATT      50

                                 60         70         80         90        100
S. tribulatum A    51 GAAGGCTGGT ATGAATGGTT GGATGAGGTA CAAGCTGTGT CATAAAAATT     100
S. tribulatum B    51 GAAGGCTGGT ATGAATGGTT GGATGAGGTA CAAGCTGTGT CATAAAAATT     100

                                110        120        130        140        150
S. tribulatum A   101 AATATTTGAA TTTAACTTTT TAGTCAAAAG GCTAAAATGT AATTAAAAGA     150
S. tribulatum B   101 AATAATTGAA TTTAACTTTT TAGTCAAAAG GCTAAAATTT AATTAAAAGA     150

                                160        170        180        190        200
S. tribulatum A   151 CGAGAAGACC CTATAGAGCT TTATATAGTT GATATTTAAT TTATTAAGAT     200
S. tribulatum B   151 CGAGAAGACC CTATAGAGCT TTATATTACA AATATTTAAT TTATTAAGAT     200

                                210        220        230        240        250
S. tribulatum A   201 TTATTAAATT TAATTATTTT ACTGTATTTT GTTGGGGTGA CAATAAAATT     250
S. tribulatum B   201 TTATTAAAAT TAATTATTTT GTTTTATTTT GTTGGGGTGA CAATAAAATT     250

                                260        270        280        290        300
S. tribulatum A   251 TATAAAACTT TTATTATTAT TTAACATTTA TTTATGGTTA TATGATCCAG     300
S. tribulatum B   251 TATAAAACTT TTATTAATAT TTAACATTTA TTTATGATTA TATGATCCAG     300

                                310        320        330        340        350
S. tribulatum A   301 TTTTATTGAT TATAAATTTA AGTTACCTTA GGGATAACAG CGTAATTTTT     350
S. tribulatum B   301 TTTTATTGAT TATAAATTTA AGTTACCTTA GGGATAACAG CGTAATTTTT     350

                                360        370        380        390        400
S. tribulatum A   351 TTTGAGAGTT CATATCGACA AAAAAGATTG CGACCTCGAT GTTGGATTAA     400
S. tribulatum B   351 TTTGAGAGTT CATATCGACA AAAAAGATTG CGACCTCGAT GTTGGATTAA     400

                                410        420        430        440        450
S. tribulatum A   401 GAGTAATTTT GGGTGTAGAA GTTCAAAGTT TAAGTCTGTT CGACTTTTAA     450
S. tribulatum B   401 GAGTAATTTT GGGTGTAGAA GTTCAAAGTT TAAGTCTGTT CGACTTTTAA     450

                                460        470        480        490        500
S. tribulatum A   451 ATTCTTACAT .......... .......... .......... ..........     500
S. tribulatum B   451 ATTCTTACAT .......... .......... .......... ..........     500
```

Technical Appendix Figure 4. Sequence of the 2 alleles obtained from amplification of a portion of the mitochondrial 16s rRNA gene from *Simulium tribulatum* infected with *Onchocerca lupi*.
